# Penile Lichen Sclerosis: A Surgical Perspective of its Aetiology and Treatment

**DOI:** 10.7759/cureus.28418

**Published:** 2022-08-26

**Authors:** Dhiraj Bhambhani, Suresh Bhambhani, Nitin Kumar Pandya

**Affiliations:** 1 Medical School, People’s College of Medical Sciences, Bhopal, IND; 2 Internal Medicine, Chirayu Medical College and Hospital, Bhopal, IND; 3 Dermatology, Gandhi Medical College, Bhopal, IND

**Keywords:** inflammatory dermatitis, management, circumcision, etiopathogenesis, penile lichen sclerosis

## Abstract

Penile lichen sclerosis is a longstanding inflammatory disease of the skin with a controversial aetiology. Penile lichen sclerosis (PLS) is a growing, inflammatory dermatitis of the anogenital region, which involves the meatus, prepuce, penile shaft, and glans penis. Although the accurate aetiology of PLS is contentious, multiple factors including genetics, autoimmunity, infections of human papillomavirus, hepatitis C, Epstein-Barr virus, risk factors (hormonal and trauma), etc., can be considered to be a part of the etiopathogenesis of PLS. The initial clinical presentations of penile lichen sclerosis are white plaques, atrophied skin, erythema, erosions, and sclerosis in the anogenital region. When the disease advances, the following can occur, including meatal constraints, telangiectasia, petechiae, soreness, papular lesions, tightness of the foreskin, difficulties in passing urine, itching, tenderness on erections, pain, cracking, bleeding, redness, rashes, tightness at frenulum, and dysuria. This disease has a dangerous course of action and if untreated it may be linked with severe urologic and sexual morbidities. PLS is usually treated with medical and surgical interventions like topical or intralesional steroids and circumcision. The role of circumcision is very critical in the course of action and prognosis of PLS, and its treatment is dependent on the stage of the disease. This review brings up the knowledge regarding epidemiology, etiopathology, clinical presentation, and management of PLS with an emphasis on the role of circumcision.

## Introduction and background

Penile lichen sclerosis (PLS) is an intensifying, inflammatory dermatitis of the anogenital region with an ambiguous aetiology; the meatus, prepuce, penile shaft, and glans penis are the commonly involved sites of the disease [1, 2]. It can be distinguished as a depigmented lesion that leads to fibrosis and obstinate soreness, provoking vicious scarring [3]. Balanitis xerotica obliterans (BXO) is a long-standing lymphocyte-mediated skin problem that occurs in the anus and genital tract areas in both men and women. In 1887, Hallopeau described this illness clinically and named it lichen plan atrophic. Then, in the year 1892, Darier called it lichen plan sclerux. In 1928, Stuhmer was the first to describe the male version of lichen sclerosis, called BXO. The BXO has three components; balanitis, which is described as long-standing swelling and tenderness of the glans penis; xerotica, which is an unusually arid exterior of the graze; and obliterans, which points toward its connection with sporadic endarteritis. In 1976, the International Society for the Study of Vulvovaginal Disease formally accepted lichen sclerosis to define this disease in both genders [3-5].

Penile LS is most commonly seen in patients aged 30-49 years, but it has also been observed in children and the elderly [3,6]. Men are usually diagnosed with lichen sclerosis at a younger age, with a peak between the ages of 30 and 50 [7]. Although LS can appear at any age, it has a bimodal age prevalence, with the highest point in adolescent boys and mature men, and a delay in the fourth decade, with female peaks occurring at prepubertal and postmenopausal ages [8,9].

The aetiology of penile lichen sclerosis remains controversial, but various factors are considered to contribute to the etiopathogenesis of penile lichen sclerosis, including genetics, autoimmunity, infections, trauma, and hormonal factors [10]. PLS causes sensitive and long-lasting balanoposthitis and discolouration due to trauma that may lead to significant sexual and urinary dysfunction. Lichen sclerosis is also associated with the risk of malignant transformation in men, mainly penile squamous cell carcinoma [11,12]. In males, lichen sclerosis has a destructive or violent path, starting initially as a scratchy scrape with pale staining on the interior side of the glans and foreskin. The progression of PLS in glans begins gently or as a dappled area, but gradually the patches merge, and the affected skin begins to lose elasticity and becomes brittle and tender rather than itchy [13]. A study by Depasquale et al. found that in a typical case of lichen sclerosis, involvement in the foreskin and glans were seen in 57% of patients, whereas the involvement of the urethra and meatus was seen in 20% and 4% of patients, respectively [14].

When the disease progresses, phimosis as well as difficulties in penile erection and sexual intercourse occur due to disfigurement of the glans penis and the foreskin. In severe cases, meatal deterioration and structural disfigurement of the glans and coronal sulcus occur. Meatal stenosis occurs due to disfigurement around the meatus, and when it spreads, it involves the fossa navicularis, penile urethra, and urethral stricture to a significant extent, whereas the bulbar urethra is rarely involved [15]. As per a study, BXO cases with urethral involvement presented with the key symptom of urethral discharge, and only one-third of the cases presented with dysuria and obstructive voiding symptoms [7]. It is established that risk is always involved in the transmission of LS to the cancerous lesion, and it is chiefly associated with female lichen sclerosis [3]. According to a study, the risk of transformation of female lichen sclerosis to squamous cell carcinoma of the vulva has been confirmed to be approximately 5% in women [3]. In males, the risk of malignant transformation of PLS is about 0.00-8.40%, which is most commonly penile squamous cell carcinoma (SCC) [16,17].

Lichen sclerosis is characteristically an enduring or lifetime disease after it begins in childhood, and no eternal diminution of the disease in puberty is possible. Sometimes LS shows reduced activity in puberty, but lichen sclerosis hardly ever goes into entire diminution [3]. The choice of treatment for PLS is medical and surgical intervention via topical or intralesional steroids and circumcision. Circumcision plays a crucial role in the emergence as well as a treatment option for lichen sclerosis. When we talk about the role of circumcision in male lichen sclerosis, it is found a major prompting factor of PLS in boys is the lack of circumcision [18]. According to some studies, the incidence of balanitis is almost double in uncircumcised boys [19-22]. The exact incidence and prevalence of PLS are difficult to identify because of the multiple areas of expertise involved in its management [23]. But the prognosis of PLS is quite good and treatment is also more successful when it is diagnosed at an early stage, and in 90-100% of cases, a potentially lasting cure is found after three months of steroid treatment or complete circumcision. In this evaluation, we will discuss the epidemiology, etiopathogenesis, clinical appearance, and management of PLS with an emphasis on the role of circumcision.

## Review

To write this review, data searching was conducted on MEDLINE (Medical Literature Analysis and Retrieval System Online), PUBMED, Google Scholar, and COCHRANE for all relevant papers and books by using various terms in the search engine like "lichen sclerosis or balanitis xerotica obliterans" or "etiopathogenesis," "autoimmunity," "genetic," "clinical presentation," "diagnosis," "male genital sclerosis," "sign and symptoms," "steroid," "epidemiology", "incidence," "prevalence," "tacrolimus," "risk factors," "malignancy," or "squamous cell carcinoma" etc.

Epidemiology

There are multiple areas of proficiency, including dermatology, genitourinary medicine, urology, and pediatrics, involved in the management of PLS. Because lichen sclerosis is frequently associated with significant under-detection and under-reporting by patients and their physicians, determining the precise incidence and prevalence of penile lichen sclerosis is difficult. However, according to a study, the predictable occurrence of lichen sclerosis varies from 1/300 (0.3%) to 1/1000 (0.1%) in both genders [23]. Regardless of the reality that this disease affects males and females both, the proportion of women to men as per available data is reported as approximately 3:1 to 10:1 [24]. As per a recent report, the frequency of genital sclerosis in males was 1.4 cases per 100,000 appointments [25]. According to one study, the prevalence of lichen sclerosis in females was 1.7% [26], indicating that the prevalence of lichen sclerosis is significantly higher in females than in males. Men developed lichen sclerosis at a younger age, with a peak between the ages of 30 and 50 [7,27]. Despite the fact that LS can appear at any age, there is a typical bimodal age prevalence in LS, with peaks in young men and grown-up men, delayed in the fourth decade, and female peaks occurring at prepubertal and postmenopausal ages [8,9]. As per research, lichen sclerosis is 10 times more prevalent in women than in men. It is predictable that the commonness of lichen sclerosis ranges from one in 30 menopausal women to one in four cases who present with professional expertise in vulval clinics, although most of the disease is misdiagnosed due to under-reporting by patients [28].

According to a study by Kizer et al., the incidence of penile lichen sclerosis was 0.07% in an unselected cohort study of 153,432 cases [27]. Although the epidemiology of male lichen sclerosis may fluctuate among nation-states and different ethnic groups since it is considered predominantly a disease of those who are uncircumcised, sometimes it can persist or re-emerge after circumcision [29,30]. According to a study, it was established that African Americans and Hispanic patients had an approximately two-fold occurrence rate of lichen sclerosis proportional to Caucasians, whilst an earlier report stated that LS is widespread in Caucasian men [3,6]. As per a study conducted among 357 males, penile lichen sclerosis was found in 52 men (15%), among whom 51 were not circumcised [31]. In contrast to that, when young boys came for circumcision, LS was found to be more prevalent, among whom 34-53% of the boys presented with symptoms of phimosis with histopathology evidence. Due to the involvement of genitals in lichen sclerosis and the unwillingness of patients to communicate in order to obtain a cure, the accurate prevalence of LS is usually reported to be low [32,33].

The Possibility of Malignant Transformation

Even though estimates vary, the risk of SCC development from LS is roughly 3%-6% in females and 2%-8% in males [14,34,35]. According to some research, the threat of cancerous transformation of penis lichen sclerosis is about 0.00-8.40%, which is most commonly penile squamous cell carcinoma (SCC) [16,17]. In Europe and North America, the prevalence of penile carcinoma is remarkable, with a frequency of less than one case per 100,000 men. In patients who were diagnosed with penile squamous cell carcinoma, lichen sclerosis was histologically illustrious in 28-44% of cases [16,36]. Although it is not very common, according to some case reports, LS patients can also develop verrucous carcinoma, basal cell carcinoma, and malignant melanoma [37,38]. According to a study, 5.8% of patients are associated with malignant transformation of penile lichen sclerosis, and the most widely spread severe transformation is penile SCC [39].

Etiopathogenesis

The exact cause of lichen sclerosis is unknown, but it is likely caused by a combination of factors.

Genetic Susceptibility

When the association of genetic factors with LS was studied, it was found that familial cases had an association with HLA antigens. Female lichen sclerosis has been found in fraternal and identical twins, sisters, mothers, and daughters [40,41]. As per a descriptive cohort study, 1052 women presented with vulval LS; among them, 12% had an affirmative family unit history of lichen sclerosis. Patients with familial lichen sclerosis were seven times more likely to have autoimmune disease than patients with sporadic lichen sclerosis [40]. In contrast to that, there is modest evidence available regarding familial predisposition to lichen sclerosis in men [14,40]. The HLA (human leukocyte antigen) is associated with both females' and males' lichen sclerosis [42,43]. The HLA complex identifies a person’s vulnerability against inflammatory diseases by persuading both cell-mediated and humoral immunity, but association between the role of HLA factors and lichen sclerosis in women is more. Several studies have found that the human leukocyte antigen (HLA) DQ7, DQ8, and DQ9 are more commonly associated with LS in females [44], whereas HLA DR11 and DR12 are more commonly associated with LS in males [45].

Autoimmunity

An individual’s family history can be associated with lichen sclerosis. It was found that some organ-targeted antibodies like anti-thyroid, gastric tissue antibodies, intrinsic factor, antinuclear, and anti-smooth muscle autoantibodies have been associated with lichen sclerosis. Patients who reported having lichen sclerosis also suffer from various autoimmune diseases like vitiligo, diabetes, thyroid disease, alopecia areata, scleroderma, and rheumatoid arthritis [46-49]. It is established that female patients with lichen sclerosis are more commonly associated with autoimmune sickness. According to a study, 350 women presented with symptoms of LS, among whom 21.5% had been associated with one or more autoimmune diseases, 21% had an association with their ancestral medical history with autoimmune diseases, and 42.0% presented with autoimmune antibodies [46]. In contrast to this, the association of autoimmune diseases with LS in males is weaker as compared with females. As per a study, 35 men presented with symptoms of LS, among whom 6% showed an alliance with autoimmune disease and 19% showed a correlation of affirmative ancestral medical history with autoimmune disease [45]. In a case-referential study, a comparison was made among 73 men suffering from lichen sclerosis and 219 healthy individuals, and it was found that vitiligo (12.1% vs. 0%) and alopecia areata (12.1% vs. 1%) were considerably more frequent in patients with LS than controls. Another study found that only 3% of 58 males with LS had an individual medical record of an autoimmune disease, and 10% had an association with the closest blood relation to autoimmune disease [43].

The extracellular matrix protein (ECM1) gene mutations and lack of functional activity are also factors in the aetiology of LS [50,51]. The ECM1 gene from humans was first isolated in 1997. The ECM1 gene has various important functions within the epidermis and dermis, like control of keratinocyte differentiation and composition union of the dermis via binding to perlecan, matrix metalloproteinase-9, and fibulin. The ECM1 also controls the growth binding factor, the basement membrane and interstitial collagen fibril assembly. It encourages the propagation of endothelial cells and also initiates angiogenesis. The failure of functional ability and mutations in the ECM1 gene were noticed, which causes lipoid proteinosis and a rare autosomal recessive genodermatosis [52]. Although autoantibodies against the ECM1 gene are found in both males and females, they may lead to an incidental side effect in the aetiology of LS [51, 53]. It was found that autoreactive ECM1 is most commonly seen in people with disease durations greater than one year. It is reported that the titer of the anti-ECM1 antibody was drastically elevated in men with LS than in controls [52]. Similarly, different percentages of CD4+ and (forkhead box P3) FOXP3+ lymphocytes have a vital role in the etiopathogenesis of both female and male lichen sclerosis but have different pathogenetic pathways [54]. The pathogenic role of antibodies targeting the basement membrane zone (BMZ), i.e. BP180 and BP230, remains uncertain according to a study in one-third of patients with vulval lichen sclerosis that found an association with BMZ [55].

Infection agents

Human Papillomaviruses (HPV)

Infection with human papillomavirus (HPV) subtypes [16,18,33,51] acts as a causative agent for penile lichen sclerosis [56]. According to studies, HPV is responsible for 52% and 64% of childhood penile lichen sclerosis, respectively [57,58]. Another study said no distinction in the prevalence of male genital lichen sclerosis was found between HPV-positive and -negative samples [59]. It was also established by research that boys who underwent circumcision of the foreskin for unrelenting phimosis after their first year of life showed the absence of HPV [60]. In penile lichen sclerosis, p16INK4a can be used instead of HPV in PLS as a biomarker [61].

Epstein-Barr Virus (EBV)

A previous study found that EBV DNA was associated with female lichen sclerosis in 26.5% of 34 vulvar biopsy cases, but its role in penile lichen sclerosis is unknown [62].

Acid-Fast Bacilli and Spirochates

According to some studies conducted earlier, an association was found between acid-fast bacilli and spirochaetes with lichen sclerosis [63,64].

Borrelia Burgdorferi

Aberer et al. identified Borrelia burgdorferi in 47% (7/15) of their lichen sclerosis cases by immunoperoxidase reaction [65,66]. Some European studies using the PCR technique found a positive correlation in 0-100% of the cases [63-72]. According to a study, an association of Borrelia burgdorferi with lichen sclerosis was found in some cases detected in Europe but not in the USA. As per investigation based on serologic culture tests, immunohistochemistry, and polymerase chain reaction (PCR), conflicting results were found against the role of Borrelia in the pathogenesis of LS [73]. Acrodermatitis chronica atrophicans is a disease that has similar clinical and histological features to lichen sclerosis, caused by Borrelia burgdorferi. On the other hand, several studies reported contradictory results, which may be due to provincial distinctions in Borrelia burgdorferi and different experimental techniques used [68].

Other risk factors

Hormones

Hormone levels are significantly associated with lichen sclerosis. If vulvar LS is untreated, it results in significantly decreased serum levels of dihydrotestosterone. According to research, reduced 5a-reductase activity acts as a causative agent for LS [74]. According to a retrospective study, females who presented with the clinical presentation of lichen sclerosis were on oral contraceptives. Therefore, OCPs act as a potential risk factor for the early onset of LS in females. While the hormonal association with male lichen sclerosis is not known [75].

Trauma and Drugs

Lichen sclerosis can arise in any area that has undergone trauma such as old scars, circumcision, after vulvectomy, skin grafts, continuous friction, radiation exposure, and sunburn [76-79]. Therefore, trauma acts as a causal agent in the aetiology of PLS. According to a study, an inverse correlation was found between vulval LS and the use of beta-blockers and ACE inhibitors. But the role of drugs in penile lichen sclerosis is still unknown [80].

Exposure to Urine

According to various research results, it was established that the lichen sclerosis of males is unambiguously a disease associated with uncircumcised males. Foreskin plays an imperative function in the aetiology of male lichen sclerosis, although it can occur in circumcised males as well who have hypospadias, genital jewellery after any surgery, instrumentation, or trauma [5,8,81]. Dribbling of some amount of urine after micturition also acts as a key source in the etiopathogenesis of penile lichen sclerosis. A recent study found that 91-100% of men with lichen sclerosis dribbled, compared to 14% of controls [82]. A small drop of post-micturition urine does not make contact with the keratinized glans in circumcised males, whereas the situation is very different in uncircumcised males. Males presented with lichen sclerosis usually have an atypical urethral meatus or navicular fossa. Usually, the dribbled urine tends to gather or pool between the inner side of the prepuce and distal penis/glans, which affects the frenulum, perimeatal glans, and visceral prepuce. It further leads to inflammation and sclerosis [83-86]. But in contrast to this, a magnetic resonance spectroscopy study recommended that no association be assessed between penile lichen sclerosis and a component of urine [87].

Metabolic Diseases

According to a recent study, metabolic or lifestyle-related diseases play an imperative role in the aetiology of male lichen sclerosis. It was observed that smoking, diabetes mellitus, high body mass index, and coronary artery disease (CAD) all directly and indirectly contribute to the etiopathogenesis of lichen sclerosis. According to a study, obese men are repeatedly presented with lichen sclerosis of the glans because of a pseudo space created by suprapubic fatness and withdrawal of the penis into the pubic mound [88,89].

Clinical presentation

Penile lichen sclerosis (LS) is an inflammatory dermatitis of the anogenital region with an indecisive aetiology. It has a dangerous course of action, and if untreated, it may be associated with significant urologic and sexual morbidities. The initial clinical presentation of LS in both men and women includes white plaques, atrophied erythematous skin, attrition, and sclerosis in the anogenital region [30,90]. Signs and symptoms of lichen sclerosis include pallor, itching meatal stricture, telangectas, petechiae, ulcers, papular lesions, bleeding, tensed foreskin, tight frenulum, dysuria, difficulty in passing urine, tenderness on erections, pain, cracking, redness and rash [2]. The usual spectrum of male lichen sclerosis is wide, which often can lead to significant morbidity. Sometimes it doesn't display any symptoms, but most of the time it looks like the preputial and urethra aren't working right, which causes male dyspareunia [8,12,91].

Some patients also display classic clinical symptoms of impulsive itching, burning, hemorrhage, scratching, and tearing of the foreskin and frenulum, which are associated with blood-filled blisters that lead to urinary problems such as pain and difficulty in urinating, anatomical alterations of the genitalia, and contraction of the urinary stream. Although the majority of men (55%) struggle with sexual activity, pain during intercourse (dyspareunia) is even more common [8]. Other symptoms of male lichen sclerosis include phimosis (the foreskin becomes non-retractile), paraphimosis (the foreskin becomes rigid in withdrawal), urethritis, urethral stricture, urine withholding, and, eventually, renal failure. This disease and its associated obstacles have a significant impact on male sexual health [8,92].

Investigation/diagnosis

The identification of penile lichen sclerosis typically depends on the clinical presentation of diseases, in which a correlation is established between changes in the paleness of skin in anogenital areas and typical itching points. According to some studies, when typical clinical characteristics are described by patients, there is no need for histopathological examination or biopsy. Although disease diagnosis is difficult in the early stages [93], normally, third men are presented with sufficient signs and symptoms for the clinical diagnosis of LS, but when the clinical picture is not leading to a decision or result, a histologic examination is advisable [51]. A genital punch biopsy is recommended for the diagnosis of lichen sclerosis. This is a simple, painless procedure that is done under local anaesthesia. It is not suitable for children [13].

The biopsy samples of lichen sclerosis illustrate some characteristic changes. In the early stages, profound infiltration of lymphocytes was seen in the basal and superficial epidermis with basal vacuolar erosion. Hyperkeratosis was also noticed in the epithelium with sclerosis and degeneration in the subepithelial collagen [3]. In the underlying dermis, the evidence of endarteritis obliterans is usually seen, which captures small arteries and arterioles [7]. After this, in the papillary dermis, there is an additional loss of elastic fibres and lymphocytic infiltrate occurs, which is shifted or replaced by sub-epidermal oedema. Further, this space is again substituted by distinguishing fibrosis. The white spot that is typical of lichen sclerosis is caused by hyperkeratosis in the epithelial base membrane and the collagen fibres that surround it [4,94].

It should be worthwhile to rule out additional comparable genital problems like lichen planus and subclinical in situ or invasive squamous cell carcinoma. The skin biopsy increases the precision of analysis and reinforces a long-term supervision plan for male lichen sclerosis [13]. Moreover, biopsy is also advisable during the routine follow-up period to rule out any misgiving of neoplastic alterations; rule out any areas which are resistant to treatment; is there any emergence of extragenital lichen sclerosis; development of any pigmented areas like the abnormal proliferation of melanocytes; etc [23,95]. Again, a biopsy is recommended to rule out another differential diagnosis of lichen sclerosis, including lichen planus (LP), lichen simplex chronicus, vitiligo, immunobullous disorders, and vulvar or penile intraepithelial neoplasia. Some of the main differential diagnoses of lichen sclerosis are lichen planus (LP), lichen simplex chronicus, vitiligo, immunobullous mucous membrane disorders such as pemphigoid and vulvar, and penile intraepithelial neoplasia [96,97].

Treatment modalities

Before deciding on available treatment choices for lichen sclerosis, it is necessary to know the course of action, prognosis, and role of circumcision in penile lichen sclerosis. As lichen sclerosis is characteristically an enduring or lifetime disease, after it begins in childhood, no eternal diminution of the disease in puberty can be possible. Sometimes LS shows reduced activity in puberty; nevertheless, lichen sclerosis hardly ever goes into complete diminution [3]. In contrast to this, according to Kirtschig et al., in men, the prognosis of PLS is quite good and treatment is also more successful. When LS is diagnosed at an early stage, permanent cutback of disease is expected in 90-100% of cases after three months of steroid treatment or complete circumcision [24]. According to a survey of 15.2 months of follow-up, it was found that among men with genital lichen sclerosis, 59% were symptom-free when treated medically (topical steroids) and with adjunctive measures. When conventional medical treatment failed, then circumcision was recommended and was curative in 76%, and a total of 24 circumcisions were required only in 24% of cases [8].

Medical Management of Lichen Sclerosis

The medical management of lichen sclerosis with mild to moderate cases includes the usage of steroids in the form of topical or intralesional injections [14]. Topical steroids are beneficial when LS is localised to the prepuce, glans, and meatus, not including noteworthy involvement and obtrusive voiding symptoms. Medical management is also helpful as an adjunct after the recurrence of surgical treatment. Topical steroids come in a variety of strengths, including 0.05% clobetasol, triamcinolone, 2.5% hydrocortisone, 0.01% mometasone, and 0.05% betamethasone, with varying success rates [98-100].

Clobetasol propionate 0.05% gel or emulsion application one to two times daily for one month is advisable [24,30], whereas some clinicians supported long-term usage of topical corticosteroids. Some authors also suggested off-label utilisation of calcineurin inhibitors like 0.1% tacrolimus, 0.1% and pimecrolimus as an alternative treatment adjunct to corticosteroids [101,102]. According to some researchers such as Dalziel et al., Sinha et al. and Cooper et al. (2004) the application of topical steroids can alleviate the indicators and occasionally resolve the clinical signs as well [103-105].

A study conducted by Riddell et al. established that the majority of men who used topical steroids as first-line therapy, and only 12% of men necessitated circumcision [2]. According to Edmonds et al., usage of steroids has shown improvement in 41%-76% of cases of LS [8]. In a study conducted in Sweden, it is documented that 30% of men said that the effect of surface steroid treatment was "good," while 37% said it was "medium," and 16% said it was "poor" for the treatment of male lichen sclerosis [92].

According to reports, long-term use of topical steroids is associated with many risk factors, such as contact sensitivity, cutaneous degeneration, and adrenal suppression. Some patients also reported disease recurrence despite topical steroids and required additional interventions [14]. In contrast to these findings, according to a study, when only steroids were used as a treatment modality for LS, recurrence occurred after five years [24]. Some studies have also found that steroid preparations can only slow disease progression but not alleviate the majority of LS cases [8,106]. According to an Indian study, among men aged between 20 and 45 years with lichen sclerosis, the first choice of treatment was circumcision rather than the use of steroid preparations [107]. In an additional Indian study, 77% of males diagnosed with PLS received circumcision for treatment [108].

Intralesional injections also give valuable results. One intralesional injection therapy is adalimumab, which is a TNF-alpha binding agent utilised for the treatment of various autoimmune diseases. Several investigators have reported that intralesional injection of adalimumab is required to prevent recurrence as well [109,110]. According to a study, polydeoxyribonucleotide injection into the intradermal site of penile tissue has an important role in enhancing the eminence of life and improving the local symptoms. It proves that the use of intralesional steroids in people who can't get relief from topical steroids is an important part of treating LS before it affects the urethra [111,112].

Surgical Treatment of Penile Lichen Sclerosis

The surgical treatment of penile lichen sclerosis usually involves circumcision, which improves symptoms and is recommended for cure in many cases. Penile lichen sclerosis starts as preputial and progresses to urethral stricture disease. The ideal choice of action for before-time lichen sclerosis is undoubtedly lingering on circumcision with or without steroids. It was shown that circumcision gives a long-term cure for about 92% of cases associated with preputial involvement [14]. When patients report the involvement of the meatal region (but not the proximal urethra), meatotomy is performed followed by the application of topical steroids, which can stop the progression of the disease and prevent future urethral involvement [113]. When the meatus and fossa navicularis were more thoroughly involved in some patients, extended meatotomy was recommended, which yielded successful results by resolving voiding symptoms in approximately 85% of patients [69].

Although in some cases, steroids are able to manage the symptoms of disease, when phimosis occurs, circumcision is commonly required. Again, when penile lichen sclerosis is allied with carcinoma in situ or invasive cancer, biopsy and/or circumcision must be executed.

When lichen sclerosis is associated with glans, circumcision is the preferred method of treatment in the majority of cases because it eliminates the moisture-rich environment that can promote the disease's recurrence. According to a large case series study which was conducted among 287 patients, circumcision was the choice of treatment when lichen sclerosis was limited to the foreskin or glans, and it was observed that 92% of patients had a termination of all their symptoms, and no additional progression of the disease was found after surgery [24].

The Role of Circumcision

The removal of the foreskin and tissues that wrap the head of the penis is called circumcision. In the olden days, circumcision was performed for religious reasons, but nowadays, many parents get their sons circumcised for various reasons, including religion, culture, medical and so on [114,115]. As per reports of various studies, approximately 25-33% of the world's males are circumcised [116-118]. When we compared the frequency of circumcision, it was 70% in the USA, 87% in Nigeria, and 6% in Britain [116,119].

When we talk about the role of circumcision in male lichen sclerosis, it is found that not performing circumcision acts as a major prompt factor in the aetiology of penile lichen sclerosis [18]. According to some studies, the incidence of balanitis is almost doubled in uncircumcised boys [20-22]. A study was conducted to check the accumulation of bacteria (E. coli, Proteus spp. and Klebsiella spp.) in boys, and it was found that the accumulation of bacteria was higher in swabbed samples pre-circumcision, but subsequent to circumcision, bacterial cultures were negative in almost 66% of the cases [120]. Nearly similar findings were derived from a Turkish study. They found 72% bacterial growth in 78 boys aged one month to 14 years before circumcision, whereas after circumcision it was only 10% [121]. Although lichen sclerosis is a multifactorial disease, lack of circumcision is a major cause associated with balanitis in men. It was established that the frequency of balanitis was accounted for among 11%-13% of men who did not undergo circumcision but only among 2% of circumcised men [31]. The results of a three-year long-term review showed that men aged 16-95 years (mean age 47 years) in Edinburgh, UK, were identified with non-specific balanitis in 22% of patients. When the status of circumcision was checked, 53% were not circumcised and only 18% were circumcised [122]. According to a retrospective study, which was conducted among 287 patients with BXO, circumcision alone was performed as a treatment modality, and on 14 years of record, 92% of patients did not entail any additional intrusion and reported lessening of their associated symptoms [14]. As per a report, circumcision is also a choice of treatment even when disease extends to the glans penis [122].

In contrast to these findings, according to a study conducted among 313 patients who went through circumcision and their specimens sent for histopathological examination, a recurrence of lichen sclerosis was found in 199 (63.6%) cases [123]. According to another study, the curative rate of circumcision was 76%, but 24% of cases required ongoing medical therapy as well, regardless of circumcision [8]. In a cohort study of previously diagnosed lichen sclerosis cases, 65-93.5% of those who had undergone circumcision had evidence of lichen sclerosis on histopathology examination [124]. As per a report that obstruction of skin in the genital can act as a potent source in the etiopathogenesis of male lichen sclerosis, it is reported that circumcised males present with fewer chances of LS, but recurrence can occur after circumcision also [31]. Circumcision is usually suggested following the failure of topical steroid treatment, chiefly in early-stage and unfussy cases [125], despite the fact that, according to some studies, long-term recurrence rates are also associated with circumcision. So, during surgery, a biopsy is recommended to rule out any kind of cancer [30].

Various problems were found in men with lichen sclerosis after performing circumcision, including meatal stenosis, urethral strictures, and phimosis. According to cohort studies, 7-20% of boys undergo circumcision for lichen sclerosis and consequently require meatal procedures like meatotomy or meatoplasty in a few weeks to months [126-130]. Almost analogous outcomes were found by Arena et al., who used uroflowmetry (UF) to appraise the conclusion of circumcision done on 75 patients with lichen sclerosis and found that 13.3% had pathological uroflowmetry [131]. One school of thought regarding circumcision says it has an imperative role in the prevention of penile lichen sclerosis, whereas the other says multiple factors are involved in the aetiology of PLS. According to some studies, circumcision is a good choice of treatment for PLS, whereas other studies say that recurrence of lichen sclerosis can occur after circumcision as well. Therefore, since the role of circumcision is not very clear due to a deficiency of conclusive evidence, long-term follow-up studies are needed to give a better conclusion and fill this gap.

## Conclusions

To summarize this review, we found that the aetiology of lichen sclerosis is still controversial, although various pieces of evidence are available that support genetics, autoimmune issues, various infectious agents, trauma, hormones, metabolic disorders, and dribbling of urine as the aetiology of lichen sclerosis. Although it is not very frequent, LS patients sometimes develop verrucous carcinoma, basal cell carcinoma, and malignant melanoma. PLS is a form of inflammatory dermatitis that has a dangerous course of action, and if untreated, it may be associated with momentous urologic and sexual morbidities. The early medical presentation of PLS includes white plaques, atrophied erythematous skin, attrition, and sclerosis in the anogenital region. When the disease progresses, patients suffer from impulsive itching, burning, haemorrhage, scratching, and tearing of the foreskin and frenulum, which are associated with blood-filled blisters that lead to urinary problems such as pain and difficulty urinating, anatomical alterations of the genitalia, and contraction of the urinary stream. The diagnosis of PLS is usually made from clinical presentation, but when the clinical picture is not very clear, a histologic examination is recommended. The prognosis is reported quite well, and treatment is also more successful when it is diagnosed at an early stage.
